# M2 Macrophage Subpopulations in Glomeruli Are Associated With the Deposition of IgG Subclasses and Complements in Primary Membranous Nephropathy

**DOI:** 10.3389/fmed.2021.657232

**Published:** 2021-05-21

**Authors:** Wenxue Hu, Guanglan Li, Jieshan Lin, Wei Dong, Feng Yu, Wei Liu, Yanhua Wu, Wenke Hao, Xinling Liang

**Affiliations:** ^1^Department of Nephrology, Guangdong Provincial People's Hospital, Guangdong Academy of Medical Sciences, Guangdong Provincial Geriatrics Institute, Guangzhou, China; ^2^Shantou University Medical College, Shantou, China; ^3^Department of Nephrology, Blood Purifiction Center, Zhongshan People's Hospital, Zhongshan, China

**Keywords:** M2 macrophages, M2 subpopulations, primary membranous nephropathy, complement, immunoglobulin

## Abstract

**Objectives:** The role of M2 macrophages in the pathogenesis and progression of primary membranous nephropathy (PMN) remains unknown. In this study, we aimed to investigate the relationship between M2 subsets and clinicopathological features of patients with PMN.

**Methods:** A total of 55 patients with PMN confirmed by biopsy were recruited. The clinical and pathological data were recorded, respectively. Immunohistochemistry was used to detect the markers of M2 macrophages, including total macrophages (CD68+), M2a (CD206+), M2b (CD86+) and M2c (CD163+).

**Results:** The numbers of glomerular macrophages, M2a, M2b, and M2c macrophages were 1.83 (1.00, 2.67), 0.65 (0.15, 1.15), 0.67 (0.33, 1.50), and 0.80 (0.05, 2.30) per glomerulus, respectively. Higher number of glomerular macrophages was found in stage II compared with stage III (2.08 vs. 1.16, *P* = 0.008). These macrophages also were negatively correlated with serum albumin level (*r* = −0.331, *P* = 0.014), while positively associated with complement 3 (C3) deposition (*r* = 0.300, *P* = 0.026) and the severity of glomerulosclerosis (*r* = 0.276, *P* = 0.041). Moreover, glomerular M2a macrophages were significantly correlated with the deposition of C3 (*r* = 0.300, *P* = 0.026), immunoglobulin G1 (IgG1) (*r* = 0.339, *P* = 0.011), immunoglobulin G2 (IgG2) (*r* = 0.270, *P* = 0.046) and immunoglobulin G3 (IgG3) (*r* = 0.330, *P* = 0.014) in glomerular basement membrane (GBM). In addition, M2b macrophages were positively associated with IgG1 (*r* = 0.295, *P* = 0.029) and IgG2 (*r* = 0.393, *P* = 0.003), while M2c macrophages were negatively correlated with complement 4d (C4d) (*r* = −0.347, *P* = 0.009) in GBM.

**Conclusions:** Our results showed that M2 macrophage subpopulations in glomeruli are associated with the deposition of IgG subclasses and complements in renal tissue of PMN, which indicate that M2 macrophages may be involved in the pathogenesis and progression of PMN. Moreover, M2a and M2c macrophages might show different tendencies in the pathogenesis of PMN.

## Introduction

Membranous nephropathy (MN) is the most common cause of nephrotic syndrome in adults. Renal biopsy typically reveals diffuse glomerular capillary wall thickening and subepithelial and/or intramembranous immune deposits. About 80% of cases are renal limited (primary MN, PMN) and 20% are associated with other systemic diseases or exposures (secondary MN). PMN is characterized by the “rule of third.” One-third of patients enter a state of spontaneous remission, and the remaining patients continue to relapse or gradually progress to renal failure (1–4). However, the mechanism of PMN remains unknown. Some studies have shown that cell-mediated immune mechanism may be actively involved in the pathogenesis of PMN (5). And macrophages play an important role in the immune mechanism. The interstitial infiltration of macrophages may indicate the outcome of PMN (6).

Macrophages can be divided into two subtypes: pro-inflammatory phenotypes, called M1 or classical activated macrophages, which can be activated by LPS or interferon. Anti-inflammatory phenotypes, namely M2 or alternative activated macrophages, which can secrete cytokines involved in tissue remodeling and fibrosis (7, 8). Recent studies have shown that M2 macrophages can be further subdivided into three subsets (9, 10). M2a macrophages (also known as wound-healing macrophages) characterized by the expression of CD206 receptors on the cell surface can be induced by IL-4 and IL-13 and are associated with tissue repair and fibrosis (11). M2b macrophages characterized by the expression of CD86 receptors on the cell surface can be induced by exposure to IC and agonists of Toll-like receptor (TLRs) or IL-1 and can regulate immune response (12). M2c macrophages characterized by the expression of CD163 receptors on the cell surface can be induced by IL10 and transforming growth factor β and exert regulatory, anti-inflammatory and pro-fibrotic functions (13).

Some studies have demonstrated that the degree of tubulointerstitial macrophage infiltration determines the prognosis of PMN (5, 14, 15). Ferrario et al. reported that macrophages infiltrated in proliferative lesions and immune-mediated human glomerulonephritis and they concluded that macrophages were involved in glomerular injury (16). However, researches on the relationship between macrophage subpopulations in glomeruli and PMN are very limited. In this study, we analyzed the relationship between M2 macrophage subpopulations in glomeruli and clinical and pathological parameters in patients with PMN.

## Materials and Methods

### Patients

A total of 55 patients with membranous nephropathy aged 18 and over were recruited in Guangdong Provincial People's Hospital from January 2018 to July 2018. Exclusion criteria: (1) Secondary membranous nephropathy, such as diabetic nephropathy, lymphoma, renal amyloidosis and so on. (2) Patients with severe cardiovascular and cerebrovascular diseases. (3) Patients with kidney transplant. (4) Patients who lack laboratory or clinical data. For all patients, renal samples were collected by biopsy and clinical data were recorded, including sex, age, proteinuria, serum creatinine (SCr), cystatin C, blood urea nitrogen (BUN), albumin and estimated glomerular filtration rate (eGFR) calculated using the Chronic Kidney Disease Epidemiology collaboration equation (CKD-EPI) at the time of biopsy. The study involving human participants was approved by the Ethical Committee of Guangdong Provincial People’s Hospital. Written informed consent was obtained from the patients before the enrollment.

### Renal Biopsies

All renal samples were obtained by percutaneous biopsy. The diagnosis of PMN is based on routine light (LM) and immumofluorescence (IF) microscopy examination. Histological staging of MN adopts the criteria proposed by Ehrenreich and Churg (17). The severity of tubular atrophy and interstitial fibrosis was rated on a scale of 0, 1 or 2 based on the percentage of affected tubules (<25, 25–50, >50%). The extent of glomerular sclerosis and the intensity of IF findings were evaluated semi-quantitatively on a scale of − (absent), + (mild), ++ (moderate), and + + + (severe).

### Immunofluorescence

The sections were deparaffinized with xylene and ethyl alcohol. Antigen repair was performed using EDTA (PH: 8.0). The sections were washed with PBS. The samples were permeabilized with 0.5% Triton-X 100 (10 min) and blocked with 5% bovine serum albumin (BSA) for 30 min at room temperature, and then incubated with the following primary antibodies diluted in 5% BSA at 4°C over-night: IgG (1:100, DAKO), IgM (1:100, Abcam), IgA (1:100, Abcam), C3 (1:200, Abcam), C1q (1:100, Abcam), C4d (1:200, Abcam), IgG1 (1:50, sigma), IgG2 (1:50, Sigma), IgG3 (1:50, Sigma), IgG4 (1:50, sigma), PLA2R (1:400, Abcam). The secondary antibody was Alexa Fluor 488 (1:1,000, Abcam) for 1 h. DAPI was used to stain the cell nuclei. The sections were observed under fluorescence microscope (Nikon 80i; Nikon, Tokyo, Japan).

### Immunohistochemistry

In this study, CD68 positive cells were identified as total macrophages, CD206 positive cells were identified as M2a, CD86 positive cells were identified as M2b and CD163 positive cells were identified as M2c. The sections were deparaffinized with xylene and ethyl alcohol. Antigen repair was performed using EDTA (PH:8.0). Endogenous peroxidase was blocked by 3% H_2_O_2_. The sections were washed with PBS. The sections were preincubated with 5% bovine serum albumin (BSA) for 30 min at room temperature, and then incubated with the following primary antibodies diluted in 5% BSA at 4°C over-night: rabbit anti-human CD68 monoclonal antibody (1:400, 76437S, CST), rabbit anti-human CD206 monoclonal antibody (1:400, 91992S, CST), rabbit anti-human CD86 monoclonal antibody (1:150, 91882S, CST) and rabbit anti-human CD163 monoclonal antibody (1:500, 93498S, CST). The secondary antibody was goat anti-rabbit IgG (1:1,000, ab6721) for 45 min. Peroxidase activities were applied by diaminobenzidine (DAB) and the sections were counterstained with hematoxylin. The sections were observed under microscope (Nikon 80i; Nikon, Tokyo, Japan).

### Qualitative Evaluation of Macrophages

A qualitative study was conducted in the absence of any clinical data. Under x40 microscope, CD68, CD206, CD86, CD163 positive cells were identified in 6 intact glomeruli per section. The average number of glomerular positive cells represents the number of macrophages per glomerulus.

### Statistical Analysis

Statistical analysis was performed using SPSS (version 25.0; SPSS Inc., Chicago, IL, USA), GraphPad Prism (version 8.0; GraphPad Software, Inc., La Jolla, CA, USA). *ANOVA-test* and *Mann-Whitney U-test* were used to compare continuous variables. *Spearman’s-test* was used for the correlation between macrophage subpopulations and clinical and pathologic data. *P*-value < 0.05 was statistically significant.

## Results

### Baseline of Patients With PMN

A total of 55 patients were recruited, including 33 (60%) males and 22 (40%) females. The mean age was 54.64 (22–80) years old. The average level of eGFR was 67.81 ± 27.68 ml/min/1.73 m^2^. The levels of proteinuria, SCr, BUN, cystatin C, and albumin were 5345.20 (3297.6, 10204.90) mg/day, 83.04 (70.47, 109.46) μmol/L, 5.88 (4.77, 7.52) mmol/L, 1.07 (0.85, 1.38) mg/L, and 23.70 (18.70, 28.50) g/L, respectively. Moreover, age was positively associated with SCr (*r* = 0.405, *P* = 0.002), cystatin C (*r* = 0.637, *P* = 0.000) and BUN (*r* = 0.489, *P* = 0.000), but negatively with eGFR (*r* = −0.796, *P* = 0.000). The patients’ baseline is shown in Table 1.

**Table 1:** Patients’ characteristics.

**Characteristics**	**Parameter**
Number of patients	55
**Gender**	
Male (*n*, %)	33 (60)
Female (*n*, %)	22 (40)
Hypertensive patients (*n*, %)	31 (56.4)
Diabetic patients (*n*, %)	8 (14.5)
Age (years, mean ± SD)	54.64 ± 14.41
eGFR_CKD−EPI_ (ml/min/1.73 m^2^, mean ± SD)	67.81 ± 27.68
Proteinuria [mg/day, M (1/4, 3/4)]	5345.20 (3297.60, 10204.90)
SCr [μmol/L, M (1/4, 3/4)]	83.04 (70.47, 109.46)
BUN [mmol/L, M (1/4, 3/4)]	5.88 (4.77, 7.52)
Cystatin-C [mg/L, M (1/4, 3/4)]	1.07 (0.85, 1.38)
Albumin [g/L, M (1/4, 3/4)]	23.70 (18.70, 28.50)

*eGFR, estimating glomerular filter rate; SCr, serum creatinine; BUN, blood urea nitrogen*.

All patients were in stage II or III of membranous nephropathy, and stage II was evident in 40 (80%) patients. Thirty-six (65.5%) patients had no glomerular sclerosis. Chronic renal tubular atrophy/interstitial fibrosis (affected area >25%) was found in only 2 cases (3.5%). In IF, granular IgG deposition was present in all patients. Most of them were IgG4 and IgG1, while IgG2 and IgG3 were also common. A weaker IgM and IgA were observed in 39 (70.9%) and 5 (9.1%) patients, respectively. Complement C3 and C4d were found in 49 patients (89.1%), and no C1q deposition was found in glomeruli. Only 9 (16.4%) patients did not express M-type phospholipase A2 receptors. The pathological parameters are listed in Table 2.

**Table 2:** The pathological data.

**Variables**	***n*** **(%)**
**Glomerular sclerosis**	
–	36 (65.5)
+	16 (29.1)
++	3 (5.5)
**Score of renal tubular/interstitial injury**	
0	53 (96.5)
1	0 (0.0)
2	2 (3.5)
**Stage of membranous nephropathy**	
II	44 (80)
III	11 (20)
**IgG**	
++	4 (7.3)
+ + +	51 (92.7)
**IgM**	
–	15 (27.3)
+	39 (70.9)
**IgA**	
–	50 (90.9)
+	5 (9.1)
**C3**	
–	6 (10.9)
+	8 (14.5)
++	37 (67.3)
+ + +	4 (7.3)
**C1q**	
–	55 (100)
**IgG1**	
–	21 (38.2)
+	16 (29.1)
++	17 (30.9)
+ + +	1 (1.8)
**IgG2**	
–	34 (61.8)
+	19 (34.5)
++	2 (3.6)
**IgG3**	
–	27 (49.1)
+	21 (38.2)
++	6 (10.9)
+ + +	1 (1.8)
**IgG4**	
–	3 (5.5)
+	3 (5.5)
++	10 (18.2)
+ + +	39 (70.9)
**PLA2R**	
–	9 (16.4)
+	46 (83.6)
**C4d**	
–	6 (10.9)
+	49 (89.1)

–*, absent; +, mild; ++, moderate; + + +, severe; IgG, immunoglobulin G; IgM, immunoglobulin M; IgA, immunoglobulin A; C3, complement 3; C1q, complement 1q; IgG1, immunoglobulin G1; IgG2, immunoglobulin G2; IgG3, immunoglobulin G3; IgG4, immunoglobulin G4; PLA2R, M-type phospholipase A2 receptor; C4d, complement 4d*.

### M2 Macrophage Subpopulations in Glomeruli of PMN

**Figure 1:**
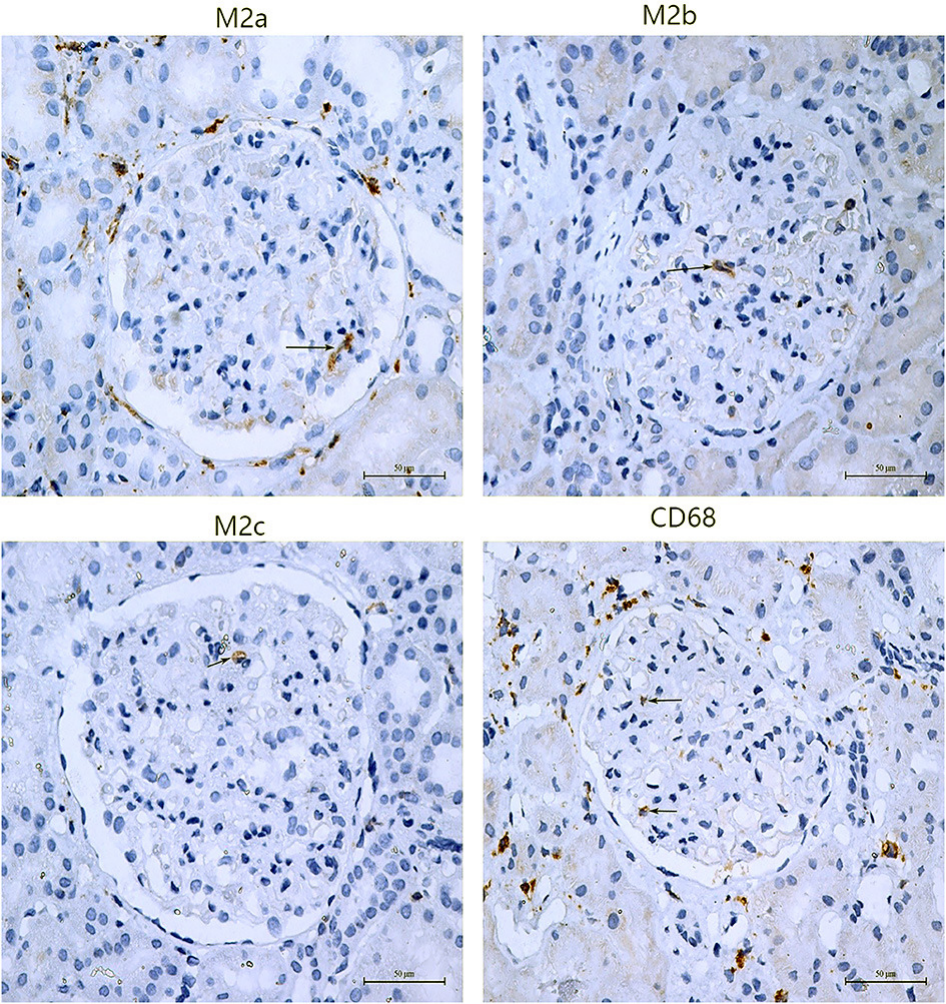
Subpopulations of M2 macrophage in glomeruli of PMN patients. M2a macrophages were positive for CD206 by immunochemistry, M2b macrophages were positive for CD86, and M2c macrophages were positive for CD163. The arrows point to positive cells.

**Figure 2:**
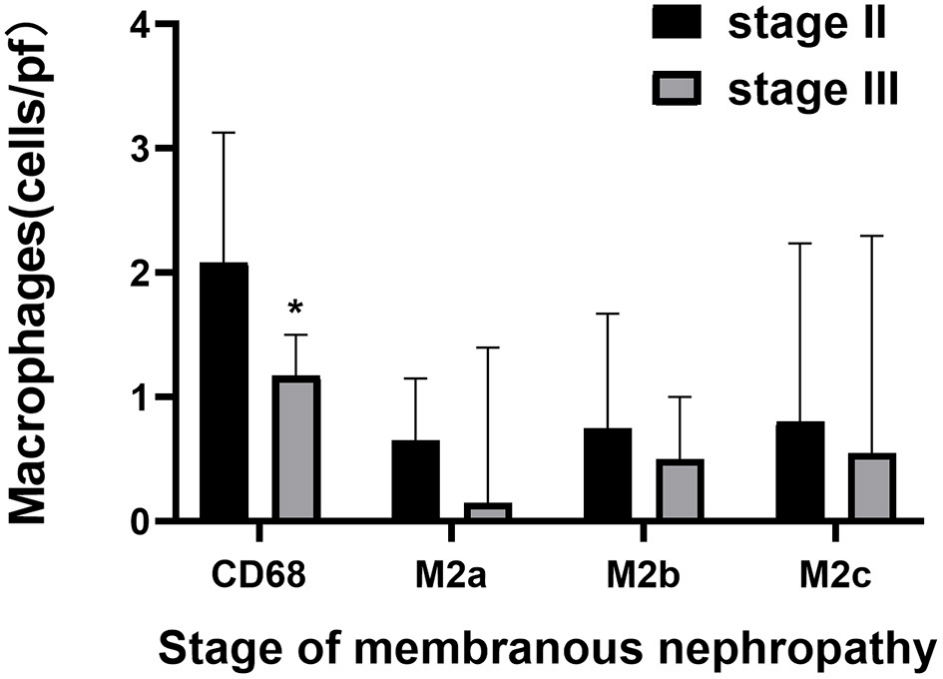
The comparison of M2 macrophage subpopulations count in glomeruli between stage II and stage III of membranous nephropathy. Quantitative analysis of CD68, M2a, M2b, and M2c in membranous nephropathy stage II and stage III, Significant correlation ^*^p < 0.05.

Macrophages and M2 macrophage subpopulations were identified by immunohistochemical staining of macrophage markers CD68, CD206, CD86, and CD163. The numbers of glomerular macrophages, M2a, M2b, and M2c macrophages were 1.83 (1.00, 2.67), 0.65 (0.15, 1.15), 0.67 (0.33, 1.50), and 0.80 (0.05, 2.30) per glomerulus, respectively. The number of M2 macrophage subpopulations in glomeruli is shown in Figure 1 and Table 3. The number of macrophages in stage II of MN was more than that in stage III [2.08 (1.04, 3.12) vs. 1.16 (0.50, 1.50), *P* = 0.008]. However, the number of M2 macrophage subpopulations was not related to the pathological staging of MN. The comparison of M2 macrophage subpopulations in glomeruli between stage II and stage III is shown in Figure 2.

**Table 3:** The numbers of M2 macrophage subpopulations.

**Variables**	***M*** **(1/4, 3/4)**
M2a	0.65 (0.15, 1.15)
M2b	0.67 (0.33, 1.50)
M2c	0.80 (0.05, 2.30)
Macrophage	1.83 (1.00, 2.67)

### The Correlation Between M2 Macrophage Subpopulations and Clinicopathological Features

We further analyzed the correlation between macrophages, M2 subpopulations and clinical data, and found that the number of macrophages was negatively associated with the level of albumin (*r* = −0.331, *P* = 0.014). However, the number of M2 macrophage subpopulations was not related to the severity of proteinuria and renal function indexes such as SCr, eGFR and cystatin C.

In terms of pathological features, macrophages count was positively correlated with C3 (*r* = 0.300, *P* = 0.026) and glomerular sclerosis (*r* = 0.276, *P* = 0.041). Moreover, M2a count was positively correlated with C3 (*r* = 0.300, *P* = 0.026), IgG1 (*r* = 0.339, *P* = 0.011), IgG2 (*r* = 0.270, *P* = 0.046), IgG3 (*r* = 0.330, *P* = 0.014), but not with IgG4 (*r* = 0.218, *P* = 0.110). M2b count was positively correlated with IgG1 (*r* = 0.295, *P* = 0.029) and IgG2 (*r* = 0.393, *P* = 0.003). For M2c, there was a negative correlation with C4d (*r* = −0.347, *P* = 0.009). The correlation between M2 macrophage subpopulations and clinicopathological data is shown in Table 4.

**Table 4:** Correlation between M2 macrophage subpopulations and clinical and pathologic data.

	**CD68**	**M2a**	**M2b**	**M2c**
eGFR_CKD−EPI_ (ml/min/1.73 m^2^)	*r* = −0.095	*r* = −0.135	*r* = −0.041	*r* = 0.060
	*P* = 0.492	*P* = 0.326	*P* = 0.767	*P* = 0.664
Proteinuria (mg/day)	*r* = 0.087	*r* = 0.108	*r* = 0.035	*r* = 0.095
	*P* = 0.525	*P* = 0.432	*P* = 0.798	*P* = 0.490
SCr (μmol/L)	*r* = 0.198	*r* = 0.064	*r* = 0.040	*r* = 0.011
	*P* = 0.147	*P* = 0.645	*P* = 0.773	*P* = 0.936
BUN (mmol/L)	*r* = 0.197	*r* = 0.063	*r* = 0.021	*r* = 0.154
	*P* = 0.149	*P* = 0.649	*P* = 0.880	*P* = 0.261
Cystatin-C (mg/L)	*r* = 0.069	*r* = 0.099	*r* = 0.021	*r* = −0.065
	*P* = 0.618	*P* = 0.473	*P* = 0.879	*P* = 0.636
Albumin	r = −0.331	*r* = −0.155	*r* = −0.167	*r* = −0.231
	*P* = 0.014^*^	*P* = 0.257	*P* = 0.224	*P* = 0.090
Glomerular sclerosis	*r* = 0.276	*r* = −0.013	*r* = 0.113	*r* = −0.134
	*P* = 0.041^*^	*P* = 0.925	*P* = 0.412	*P* = 0.329
IgG	*r* = −0.020	*r* = 0.002	*r* = −0.204	*r* = −0.038
	*P* = 0.885	*P* = 0.987	*P* = 0.135	*P* = 0.785
IgM	*r* = −0.247	*r* = −0.151	*r* = −0.218	*r* = −0.003
	*P* = 0.072	*P* = 0.275	*P* = 0.114	*P* = 0.985
IgA	*r* = −0.186	*r* = −0.006	*r* = 0.030	*r* = 0.142
	*P* = 0.175	*P* = 0.965	*P* = 0.828	*P* = 0.301
C3	*r* = 0.300	*r* = 0.300	*r* = 0.056	*r* = 0.263
	*P* = 0.026^*^	*P* = 0.026^*^	*P* = 0.686	*P* = 0.052
IgG1	*r* = 0.142	*r* = 0.339	*r* = 0.295	*r* = 0.262
	*P* = 0.300	*P* = 0.011^*^	*P* = 0.029^*^	*P* = 0.054
IgG2	*r* = 0.202	*r* = 0.270	*r* = 0.393	*r* = 0.136
	*P* = 0.138	*P* = 0.046^*^	*P* = 0.003^*^	*P* = 0.321
IgG3	*r* = 0.077	*r* = 0.330	*r* = 0.165	*r* = 0.085
	*P* = 0.575	*P* = 0.014^*^	*P* = 0.229	*P* = 0.536
IgG4	*r* = 0.028	*r* = 0.218	*r* = −0.045	*r* = 0.087
	*P* = 0.837	*P* = 0.110	*P* = 0.747	*P* = 0.528
PLA2R	*r* = −0.047	*r* = −0.058	*r* = 0.075	*r* = 0.061
	*P* = 0.736	*P* = 0.675	*P* = 0.588	*P* = 0.660
C4d	*r* = 0.081	*r* = −0.137	*r* = −0.129	*r* = −0.347
	*P* = 0.557	*P* = 0.318	*P* = 0.347	*P* = 0.009^*^

*Significant correlation*

* *p < 0.05*.

### The Correlation Between Clinical Data and Pathologic Data

**Figure 3:**
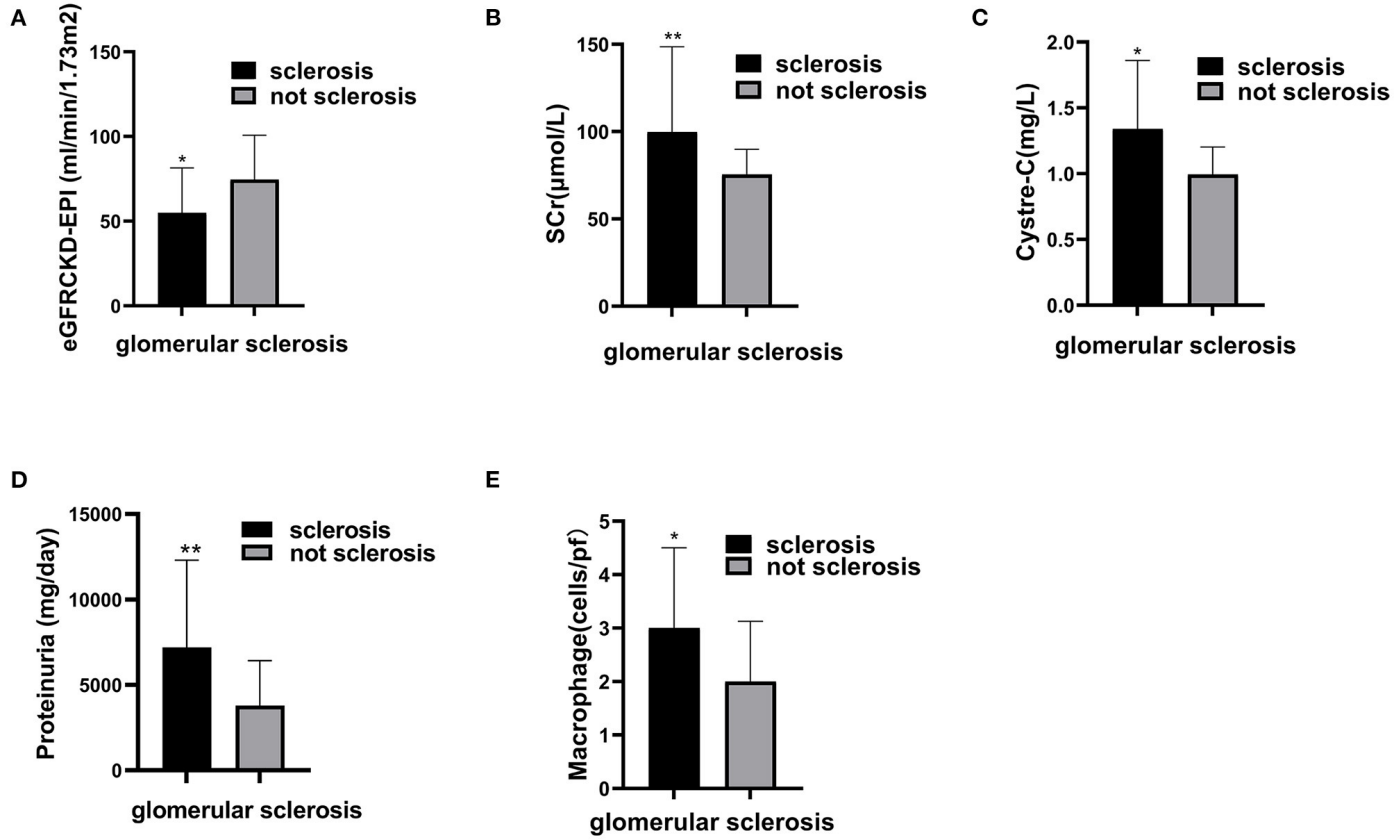
The correlation between glomerular sclerosis and clinical features and macrophages in glomeruli. Compare estimating glomerular filter rate **(A)**, serum creatinine **(B)**, Cystre-C **(C)**, Proteinuria **(D)**, and macrophage **(E)** between patients with glomerular sclerosis and patients without glomerular sclerosis. Significant correlation ^*^p < 0.05, ^**^p < 0.01.

Our data showed patients with glomerular sclerosis had poor renal function. The level of eGFR in patients with glomerular sclerosis was lower than that in patients without glomerular sclerosis (54.92 ± 26.50 vs. 74.61 ± 26.13) (*P* = 0.011). The levels of SCr and cystatin C in patients with glomerular sclerosis were higher than those in patients without glomerular sclerosis, which were 99.80 (86.47, 148.60) vs. 75.53 (67.19, 89.83) (*P* = 0.005), 1.34 (0.89, 1.86) vs. 0.99 (0.80, 1.20) (*P* = 0.014), respectively. Furthermore, the level of proteinuria in patients with glomerular sclerosis was higher than that in patients without glomerular sclerosis, which was 7191.00 (4832.00, 12292.00) vs. 3796.70 (2442.08, 6422.69) (*P* = 0.001). The correlation between clinical data and pathologic data is shown in Figure 3.

## Discussion

In this study, we identified total macrophages and M2 macrophage subpopulations in the glomeruli of patients with PMN. Correlation analysis showed that the total macrophage count was negatively associated with serum albumin level, while positively associated with C3 deposition and the severity of glomerular sclerosis. M2 subpopulations were found in glomerulus. M2a macrophages in glomeruli were significantly correlated with the deposition of C3, IgG1, IgG2, and IgG3 in glomerular basement membrane (GBM) and M2b macrophages were positively associated with the deposition of IgG1 and IgG2. M2c macrophages were negatively correlated with complement 4d (C4d).

PMN is now considered to be a renal limited autoimmune disease, with antibodies against M-type phospholipase A2 receptor (PLA2R) identified in 70–80% patients (18). In our study, 46 (83.6%) patients were identified expressing PLA2R in renal tissue, which is in accord with the previous studies. Moreover, MN is also considered to be a IgG4 dominant disease and universal presence of C3 in the subepithelial deposits (19, 20). Our results showed that IgG4 was dominant in immune complexes and complement C3 and C4d were prevalence in 89.1% of these patients. The data above indicate that our study population has representative pathological features of PMN.

Tissue macrophages are crucial players in inflammation and immunity (21). In our study, a small number of macrophages (1.83 per glomeruli) was found in glomeruli and positively correlated with complement C3 that is the main deposits of MN. Papagianni et al. showed that macrophages released cytokines, which induced consecutive complement activation leading to tubular damage in patients with membranous nephropathy. They also reported that SCr was highly correlated with the number of interstitial macrophages (22). The aforementioned evidence indicates that macrophages may be involved in the progression of MN through the mechanism of complement activation. Moreover, our results showed that the number of macrophages was positively correlated with the extent of glomerular sclerosis and negatively correlated with serum albumin. Saito and Atkins pointed out that macrophages increased in experimental and clinical focal segmental glomerular sclerosis (FSGS) (23). Morita et al. demonstrated that the level of eGFR in MN patients with FSGS was lower than those without FSGS (24). Therefore, macrophages may participate in the progress of FSGS in PMN patients.

PMN has four evolutionary stages according to the criteria proposed by Ehrenreich and Churg. In our study, all patients were in membranous nephropathy stage II or III. Papagianni et al. showed that the stage of the PMN was not related to the degree of glomerular infiltration by inflammatory cell (22). However, our results showed that more macrophages in glomeruli were found in stage II than in stage III, which may be associated with earlier immune response in stage II. But the number of M2 macrophage subpopulations was not related to the stage of membranous nephropathy. Therefore, larger samples and further studies are needed to analyze the role of macrophages in different stages of PMN.

M2 macrophages are composed of a heterogeneous subpopulation of cells with different functions and phenotypic plasticity. M2a macrophages, characterized by the expression of transmembrane marker CD206, are known to be involved in the progression of kidney disease (25). In our study, we found that M2a macrophages were positively associated with IgG1, IgG2, and IgG3. As we known, IgG4 is the predominant IgG subclass in PMN, but IgG1, IgG2, and IgG3 are also common. Recent studies suggested that there was a programmed order of immune response, starting from IgM producing B cells followed by class switching to IgG producing B cells, with production of IgG subclass antibodies in a fixed order of IgG3 > IgG1> IgG2 > IgG4 (26). Therefore, M2a macrophages may be associated with the early phase of IgG production. This hypothesis needs to be validated by further basic and clinical studies. Moreover, our data also showed that M2a macrophages were associated with C3. Previous study proved that C3 in subepithelial deposits is very common in MN (27), and IgG1 and IgG3 were associated with complement activation and fixing. These results show that M2a may be involved in the progression of PMN through promoting the deposition of IgG subtypes and activating complements.

M2b macrophages regulate immune response and are induced by immune complexes (IC). And PMN is characterized by IC. It has been reported that M2b macrophages may be a crucial mediator for the initiation and progression of autoimmune diseases (28). Our study showed that M2b macrophages were observed in PMN and were positively associated with IgG1 and IgG2. Orme and Mohan demonstrated that M2b macrophages actually play a direct role in causing SLE (29). M2b macrophages, as antigen-presenting cells, are involved in innate and adaptive immunity, and immunoglobulin is an important part of adaptive immunity, suggesting that M2b may promote the production of IgG by presenting antigen. These data above indicate that M2b might be involved in the progressionpbr of PMN.

M2c characterized by the expression of CD163 receptor on cell surface can exert regulatory, anti-inflammatory and pro-fibrotic functions. Several studies have shown that M2c macrophages have protective effects. Tseng et al. showed that the upregulation of M2c macrophages could alleviate renal fibrosis in obstructed kidney (30). Lu et al. transferred M2c macrophages into mice on day 5 after adriamycin administration, and the results showed that M2c effectively reduced glomerulosclerosis, tubular atrophy, interstitial expansion, and proteinuria, and they concluded that M2c might protect the kidney from injury (31). In addition, Tang et al. indicated that M2c macrophages could ameliorate inflammation and fibroproliferation in acute lung injury through interleukin 10 pathway (32). These results were accordant with ours. In our study, we found that C4d was associated with M2c negatively. Positive staining with C4d was present in immune-complex related glomerulonephritis, including membranous glomerulonephritis and lupus nephritis, which revealed that C4d was involved in immune reaction in PMN (33). Therefore, we speculate that M2c may play a protective role in PMN through reducing the production of C4d.

In this study, we described the association between M2 macrophage subpopulations in glomeruli and pathological features in PMN with novelty. However, our study has some limitations. Renal biopsies from human samples can only represent a snapshot of the current state of the disease. Therefore, we have no dynamic information about the early stages of the disease and the progression of macrophage subtypes. In addition, we only analyze the surface markers of M2 subtypes and other markers of M2 subtypes (such as functional cytokines) need to be analyzed to support our conclusions. We will detect more markers and follow up the patients to investigate the role of M2 subpopulations in PMN.

## Conclusions

In conclusion, our study showed that M2a and M2b macrophages were positively correlated with tissue IgG subclasses of early stage and C3, while M2c macrophages were negatively correlated with C4d. These results indicate that M2 macrophage subpopulations are involved in the progression of PMN by the deposition of IgG subclasses and complements. And M2a and M2c macrophages might show different properties in the pathogenesis of PMN.

## Data Availability Statement

The original contributions presented in the study are included in the article/supplementary material, further inquiries can be directed to the corresponding authors.

## Ethics Statement

The studies involving human participants were reviewed and approved by The Ethical Committee of Guangdong Provincial People’s Hospital. The patients/participants provided their written informed consent to participate in this study.

## Author Contributions

WHu: conceptualization, methodology, formal analysis, and writing-original draft. GL: conceptualization, data curation, investigation, and writing-reviewing and editing. JL and WL: data curation, visualization, investigation, and software. WD and YW: software, formal analysis, data curation, and investigation. FY: software, formal analysis, and investigation. WHa: conceptualization, supervision, writing-reviewing and editing, and validation. XL: conceptualization, resources, supervision, writing-reviewing and editing, and validation. All authors contributed to the article and approved the submitted version.

## Conflict of Interest

The authors declare that the research was conducted in the absence of any commercial or financial relationships that could be construed as a potential conflict of interest.

## References

[1] Davison AM Cameron JS Kerr DN Ogg CS Wilkinson PW. The natural history of renal function in untreated idiopathic membranous glomerulonephritis. Clin Nephrol. (1984) 22:61–7. 6478673

[2] Ponticelli C. Prognosis and treatment of membranous nephropathy. Kidney Int. (1986) 29:927–40. 10.1038/ki.1986.88 2940407

[3] Donadio J Torres V Velosa J Wagoner RD Holley KE Okamura M. Idiopathic membranous nephropathy: the natural history of untreated patients. Kidney Int. (1988) 33:708–15. 10.1038/ki.1988.56 3367560

[4] Francis JM Beck LH Jr Salant DJ. Membranous nephropathy: a journey from bench to bedside. Am J Kidney Dis. (2016) 68:138–47. 10.1053/j.ajkd.2016.01.030 27085376 PMC4921260

[5] Alexopoulos E Seron D Hartley RB Nolasco F Cameron JS. Immune mechanisms in idiopathic membranous nephropathy: the role of the interstitial infiltrates. Am J Kidney Dis. (1989) 13:404–12. 10.1016/S0272-6386(89)80024-1 2785756

[6] Yoshimoto K Wada T Furuichi K Sakai N Iwata Y Yokoyama H. CD68 and MCP-1/CCR2 expression of initial biopsies reflect the outcomes of membranous nephropathy. Nephron Clin Pract. (2004) 98:c25–34. 10.1159/000079924 15361701

[7] O’Neill LA Pearce EJ. Immunometabolism governs dendritic cell and macrophage function. J Exp Med. (2016) 213:15–23. 10.1084/jem.20151570 26694970 PMC4710204

[8] Tarique AA Logan J Thomas E Holt PG Sly PD Fantino E. Phenotypic, functional, and plasticity features of classical and alternatively activated human macrophages. Am J Respir Cell Mol Biol. (2015) 53:676–88. 10.1165/rcmb.2015-0012OC 25870903

[9] Martinez FO Sica A Mantovani A Locati M. Macrophage activation and polarization. Front Biosci. (2008) 13:453–61. 10.2741/2692 17981560

[10] Mosser DM Edwards JP. Exploring the full spectrum of macrophage activation. Nat Rev Immunol. (2008) 8:958–69. 10.1038/nri2448 19029990 PMC2724991

[11] Wang Y Harris DC. Macrophages in renal disease. J Am Soc Nephrol. (2011) 22:21–7. 10.1681/ASN.2010030269 21209251

[12] Sironi M Martinez FO D’Ambrosio D Gattorno M Polentarutti N Locati M. Differential regulation of chemokine production by Fcgamma receptor engagement in human monocytes: association of CCL1 with a distinct form of M2 monocyte activation (M2b, Type 2). J Leukoc Biol. (2006) 80:342–9. 10.1189/jlb.1005586 16735693

[13] Benoit M Desnues B Mege JL. Macrophage polarization in bacterial infections. J Immunol. (2008) 181:3733–9. 10.4049/jimmunol.181.6.3733 18768823

[14] Wu Q Jinde K Nishina M Tanabe R Endoh M Okada Y. Analysis of prognostic predictors in idiopathic membranous nephropathy. Am J Kidney Dis. (2001) 37:380–7. 10.1053/ajkd.2001.21319 11157381

[15] Lee AH Bass PS Williams JH Evans B Jones DB Theaker JM. CD45RO and CD45RA positive cell populations in idiopathic membranous and IgA glomerulopathy. J Clin Pathol. (1996) 49:43–7. 10.1136/jcp.49.1.43 8666684 PMC1023156

[16] Ferrario F Castiglione A Colasanti G Barbiano di Belgioioso G Bertoli S D’Amico G. The detection of monocytes in human glomerulonephritis. Kidney Int. (1985) 28:513–9. 10.1038/ki.1985.158 4068484

[17] Ehrenreich T Churg J. Pathology of membranous nephropathy. Pathol Ann. (1968) 3:145–86.

[18] Du Y Li J He F Lv Y Liu W Wu P. The diagnosis accuracy of PLA2R-AB in the diagnosis of idiopathic membranous nephropathy: a meta-analysis. PLoS ONE. (2014) 9:e104936. 10.1371/journal.pone.0104936 25136841 PMC4138154

[19] Zhang XD Cui Z Zhang MF Wang J Zhang YM Qu Z. Clinical implications of pathological features of primary membranous nephropathy. BMC Nephrol. (2018) 19:215. 10.1186/s12882-018-1011-5 30153817 PMC6114049

[20] van de Logt AE Fresquet M Wetzels JF Brenchley P. The anti-PLA2R antibody in membranous nephropathy: what we know and what remains a decade after its discovery. Kidney Int. (2019) 96:1292–302. 10.1016/j.kint.2019.07.014 31611068

[21] Chen T Cao Q Wang Y Harris DCH. M2 macrophages in kidney disease: biology, therapies, and perspectives. Kidney Int. (2019) 95:760–33. 10.1016/j.kint.2018.10.041 30827512

[22] Papagianni AA Alexopoulos E Leontsini M Papadimitriou M. C5b-9 and adhesion molecules in human idiopathic membranous nephropathy. Nephrol Dial Transplant. (2002) 17:57–63. 10.1093/ndt/17.1.57 11773463

[23] Saito T Atkins RC. Contribution of mononuclear leucocytes to the progression of experimental focal glomerular sclerosis. Kidney Int. (1990) 37:1076–83. 10.1038/ki.1990.88 2342246

[24] Morita M Mii A Shimizu A Yasuda F Shoji J Masuda Y. Glomerular endothelial cell injury and focal segmental glomerulosclerosis lesion in idiopathic membranous nephropathy. PLoS ONE. (2015) 10:e0116700. 10.1371/journal.pone.0116700 25875837 PMC4398543

[25] Tang PM Nikolic-Paterson DJ Lan HY. Macrophages: versatile players in renal inflammation and fibrosis. Nat Rev Nephrol. (2019) 15:144–58. 10.1038/s41581-019-0110-2 30692665

[26] Collins AM Jackson KJ. A Temporal model of human IgE and IgG antibody function. Front Immunol. (2013) 4:235. 10.3389/fimmu.2013.00235 23950757 PMC3738878

[27] Hoxha E Kneibler U Stege G Zahner G Thiele I Panzer U. Enhanced expression of the M-type phospholipase A2 receptor in glomeruli correlates with serum receptor antibodies in primary membranous nephropathy. Kidney Int. (2012) 82:797–804. 10.1038/ki.2012.209 22673885

[28] Wang LX Zhang SX Wu HJ Rong XL Guo J. M2b macrophage polarization and its roles in diseases. J Leukoc Biol. (2019) 106:345–58. 10.1002/JLB.3RU1018-378RR 30576000 PMC7379745

[29] Orme J Mohan C. Macrophage subpopulations in systemic lupus erythematosus. Discov Med. (2012) 13:151–8. 22369974

[30] Tseng WC Tsai MT Chen NJ Tarng DC. Trichostatin A alleviates renal interstitial fibrosis through modulation of the M2 macrophage subpopulation. Int J Mol Sci. (2020) 21:5966. 10.3390/ijms21175966 32825118 PMC7503910

[31] Lu J Cao Q Zheng D Sun Y Wang C Yu X. Discrete functions of M2a and M2c macrophage subsets determine their relative efficacy in treating chronic kidney disease. Kidney Int. (2013) 84:745–55. 10.1038/ki.2013.135 23636175

[32] Tang L Zhang H Wang C Li H Zhang Q Bai J. M2A and M2C macrophage subsets ameliorate inflammation and fibroproliferation in acute lung injury through interleukin 10 pathway. Shock. (2017) 48:119–29. 10.1097/SHK.0000000000000820 27941591

[33] Val-Bernal JF Garijo MF Val D Rodrigo E Arias M. C4d immunohistochemical staining is a sensitive method to confirm immunoreactant deposition in formalin-fixed paraffin-embedded tissue in membranous glomerulonephritis. Histol Histopathol. (2011) 26:1391–7. 10.14670/HH-26.1391 21938676

